# Self-reported life-space mobility in the first year after ischemic stroke: longitudinal findings from the MOBITEC-Stroke project

**DOI:** 10.1007/s00415-023-11748-5

**Published:** 2023-05-04

**Authors:** Timo Hinrichs, Roland Rössler, Denis Infanger, Robert Weibel, Janine Schär, Eva-Maria Peters, Erja Portegijs, Taina Rantanen, Arno Schmidt-Trucksäss, Stefan T. Engelter, Nils Peters

**Affiliations:** 1 Division of Sport and Exercise Medicine, Department of Sport, Exercise, and Health, University of Basel, Grosse Allee 6, 4052 Basel, Switzerland; 2 Basel Mobility Center, University Department of Geriatric Medicine Felix Platter, University of Basel, Basel, Switzerland; 3 Department of Geography, University of Zurich, Zurich, Switzerland; 4 University Research Priority Program (URPP) Dynamics of Healthy Aging, University of Zurich, Zurich, Switzerland; 5 Neurology and Neurorehabilitation, University Department of Geriatric Medicine Felix Platter, University of Basel, Basel, Switzerland; 6 Neurology and Stroke Center, Klinik Hirslanden, Zurich, Switzerland; 7 Center for Human Movement Sciences, University of Groningen, University Medical Center Groningen, Groningen, The Netherlands; 8 Faculty of Sport and Health Sciences and Gerontology Research Center, University of Jyvaskyla, Jyväskylä, Finland; 9 Department of Neurology and Stroke Center, University Hospital Basel and University of Basel, Basel, Switzerland

**Keywords:** Cohort studies, Spatial behavior, Mobility limitation, Physical functional performance, Social participation

## Abstract

**Background:**

Life-space mobility is defined as the size of the area in which a person moves about within a specified period of time. Our study aimed to characterize life-space mobility, identify factors associated with its course, and detect typical trajectories in the first year after ischemic stroke.

**Methods:**

MOBITEC-Stroke (ISRCTN85999967; 13/08/2020) was a cohort study with assessments performed 3, 6, 9 and 12 months after stroke onset. We applied linear mixed effects models (LMMs) with life-space mobility (Life-Space Assessment; LSA) as outcome and time point, sex, age, pre-stroke mobility limitation, stroke severity (National Institutes of Health Stroke Scale; NIHSS), modified Rankin Scale, comorbidities, neighborhood characteristics, availability of a car, Falls Efficacy Scale-International (FES-I), and lower extremity physical function (log-transformed timed up-and-go; TUG) as independent variables. We elucidated typical trajectories of LSA by latent class growth analysis (LCGA) and performed univariate tests for differences between classes.

**Results:**

In 59 participants (mean age 71.6, SD 10.0 years; 33.9% women), mean LSA at 3 months was 69.3 (SD 27.3). LMMs revealed evidence (*p* ≤ 0.05) that pre-stroke mobility limitation, NIHSS, comorbidities, and FES-I were independently associated with the course of LSA; there was no evidence for a significant effect of time point. LCGA revealed three classes: “low stable”, “average stable”, and “high increasing”. Classes differed with regard to LSA starting value, pre-stroke mobility limitation, FES-I, and log-transformed TUG time.

**Conclusion:**

Routinely assessing LSA starting value, pre-stroke mobility limitation, and FES-I may help clinicians identify patients at increased risk of failure to improve LSA.

## Introduction

Ischemic stroke is one of the main aging-related diseases [1, 2] and is a major risk factor for incident disability in activities of daily living (ADL) [3, 4]. It frequently results in permanent functional limitations [5] and—even in individuals with mild to moderate stroke—has a major impact on patients’ self-perceived mobility and participation in social life [6].

“Life-space mobility” refers to the spatial area in which people move about within their daily lives, potentially ranging from staying in the room in which one sleeps to traveling out of town. It includes the frequency of travel and assistance needed [7] and thus reflects the interplay between people’s goals, capabilities, opportunities, and demands of their environment [8, 9]. In the general older population, life-space mobility predicts disability [10], nursing home admission [11], health care utilization [12], and mortality [13]. Furthermore, life-space mobility is positively associated with quality of life [14], and its decline over time is associated with a decline of quality of life [15]. Based on its relevance for personal health and social interaction, life-space mobility can be considered an important patient-oriented outcome in geriatric rehabilitation [16].

So far, research on life-space mobility after stroke has been sparse. A longitudinal study that followed-up 89 patients post-stroke (median time after event at baseline 75 months) showed a significant decline in life-space mobility over a 2-year period [17]. After adjustment for a number of potential confounders, higher age and lower comfortable gait speed were significantly associated with a decrease in life-space mobility over time. There is also some evidence from cross-sectional studies that functional independence, lower extremity physical function, falls efficacy, and health-related quality of life are positively associated with life-space mobility after stroke [18–20]. Overall, there is a lack of prospective, longitudinal studies assessing people’s life-space mobility at several clearly defined points in time after stroke.

In our study, we aimed to (a) describe life-space mobility, (b) identify factors associated with its course, and (c) detect typical trajectories in the first year after an ischemic stroke.

## Methods

### Study design

MOBITEC-Stroke (“Recovery of mobility function and life-space mobility after ischemic stroke”; ISRCTN85999967) was a prospective observational study approved by the Ethics Committee of Northwestern and Central Switzerland (Reg.-No. 2019-00989). All participants provided written informed consent. Assessments were conducted at the research center 3 (*T*_0_), 6 (*T*_1_), 9 (*T*_2_) and 12 (*T*_3_) months after stroke. Clinical data from the time of event were retrieved from clinical records. The full study protocol is available elsewhere [21].

### Target group, inclusion and exclusion

MOBITEC-Stroke targeted community-dwelling, ambulatory patients after a first ischemic stroke. Inclusion criteria were: first ischemic stroke (confirmed by brain imaging) within the previous 3 months; age ≥ 18 years; ability to communicate verbally; ability to understand the study information and to provide written informed consent; ability to get up from a chair and sit down without help; ability to walk for a minimum of 20 m at their own pace, with or without pauses, with or without a walking aid, but without personal assistance; and presence of at least one of the following stroke-related symptoms potentially affecting gait and mobility: lower limb paralysis or ataxia, stance/gait ataxia (cerebellar or sensory), visual disturbance/field defect, central vestibular deficit or attentional deficit/neglect.

Exclusion criteria included the following: not living in one’s own home; inability to walk without assistance (modified Rankin Scale, mRS, > 3); severe cognitive impairment (Montreal Cognitive Assessment score < 21 or, for persons with ≤ 12 years of education, < 20) [22]; acute psychiatric disorder; advanced terminal illness; orthopedic surgery of the lower extremities within the previous year or on-going rehabilitation measures following an inpatient surgical procedure at the time of stroke. The following questions were used to assess pre-stroke mobility limitation: “In the week before the stroke, were you able to walk 2 km?” and “In the week before the stroke, were you able to climb 1 flight of stairs?” [23]. Response options were “Yes, without difficulty”; “Yes, but with some difficulty”; “Yes, but with a great deal of difficulty”; “Yes, but not without help”; and “Not even with help”. Those reporting at least “a great deal of difficulty” in 1 of the 2 activities were excluded from participation.

### Recruitment and participants

All patients presenting at the Stroke Center, University Hospital Basel, with an acute ischemic stroke between October 2019 and March 2021 were screened for eligibility. All eligible patients were offered the opportunity to participate in the study. Recruitment was stopped once the targeted sample size of *N* = 59 (see study protocol [21]) was reached.

### Measures

We used the University of Alabama at Birmingham Study of Aging Life-Space Assessment (LSA) to measure life-space mobility at four time points (*T*_0_–*T*_3_; 3, 6, 9 and 12 months post stroke) [7]. Participants were asked to report the extent of their movement within the previous 4 weeks, categorized into five spatial levels (1 = rooms in the house outside of the room in which they sleep, 2 = immediate outdoor area, 3 = own neighborhood, 4 = outside their own neighborhood but within town, and 5 = out of town), the frequency of traveling to these levels (1 = less than once/week, 2 = 1–3 times/week, 3 = 4–6 times/ week, and 4 = daily), and whether they needed assistance (ie, 1 = personal assistance, 1.5 = assistive devices, 2 = no assistance). First, a subscore for every level was calculated by multiplying the values (as stated in parentheses above) for level, frequency, and assistance; subscores were then added to derive the composite score (used for all analyses) ranging from 0 (completely bedridden) to 120 (visiting out-of-town places every day unassisted); ie, higher scores indicate better life-space mobility. Previous research suggests considering an LSA composite score of lower than 60 as being “restricted” in life-space mobility [11, 24]—indicating that a person generally remains at home or in their neighborhood—and a change of ≥ 5 as being clinically important [25]. LSA has been reported to be highly reliable, valid and sensitive to change [7, 26, 27].

At *T*_0_ (3 months post stroke), pre-stroke mobility limitation (ie, difficulty walking 2 km and/or climbing 1 flight of stairs in the week before the event) was assessed [23]. Participants also underwent a clinical-neurological examination to determine stroke severity (National Institutes of Health Stroke Scale; NIHSS) [28] and the level of functional independence (mRS). The presence of comorbidities (heart disease, high blood pressure, lung disease, diabetes, ulcer or stomach disease, kidney disease, liver disease, anemia or other blood disease, cancer, depression, osteoarthritis/degenerative arthritis, back pain, rheumatoid arthritis) was assessed using the Self-Administered Comorbidity Questionnaire (SCQ) (“Do you have the problem?” yes vs no) [29].

At *T*_0_ (3 months post stroke) and at all follow-up visits (*T*_1_–*T*_3_; 6, 9 and 12 months post stroke), the current type of neighborhood (urban vs suburban vs rural) [30], availability of a car (yes vs no), and the level of concern about falling when performing various activities (Falls Efficacy Scale–International Version; FES-I) [31] were assessed by self-report. FES-I scores may range from 16 (no concern about falling) to 64 (severe concern about falling). Lower extremity physical function was assessed using the timed up-and-go (TUG) test, a timed test in which the individual stands up from a chair, walks around a cone 3 m away and returns to sitting in the chair—a variation suggested by Rikli and Jones [32] as an alternative to the test in which the individual walks to a mark on the floor, turns around and walks back to the starting position [33].

### Statistical analyses

Participant characteristics (at *T*_0_; 3 months post stroke) as well as LSA (at *T*_0_–*T*_3_; 3, 6, 9 and 12 months post stroke) were analyzed descriptively (numbers/percentages or mean/SD/median/IQR, respectively).

For all further analyses, the following variables were dichotomized: age (≤ vs > median; ie, ≤ vs > 74 years); pre-stroke mobility limitation (yes = at least some difficulty walking 2 km or climbing stairs vs no = no difficulty with either activity); NIHSS (< vs ≥ median; ie, 0–1 vs ≥ 2); mRS (0–1 vs ≥ 2; ie, no symptoms or no significant disability despite symptoms vs at least slight disability), and SCQ (< 2 vs ≥ 2 comorbidities). Due to its skewed distribution, TUG time was log-transformed.

We used linear mixed effects models (LMMs) with LSA score as outcome and time point, sex, age category, pre-stroke mobility limitation category, NIHSS category, mRS category, comorbidity category, type of neighborhood, availability of a car for personal use, FES-I score, and log-transformed TUG time as independent variables. For sex, age category, mRS category and NIHSS category, interactions with time point were also included as independent variables. For sex, NIHSS category, age category, pre-stroke mobility-limitation category, and mRS category, the baseline values were used; type of neighborhood, availability of a car for personal use, FES-I score and log-transformed TUG time were included as time-varying variables. As specified in the protocol, we included time as a discrete variable [21]. The model included a random intercept for subjects and was fitted using maximum likelihood for unbiased estimation of the fixed effects. We used multiple imputation to account for missing data, which would have led to a loss of 28 (of 236) incomplete observations in the model (11.9%)—with “observation” referring to a set of data of all assessed variables of a specific participant at a specific time point [34]. Specifically, we assumed an MAR process for the missing data and imputed 60 datasets using weighted predictive mean matching for continuous variables and logistic or multinomial regression for binary or polytomous categorical variables. The R package “mice” was used for imputations. Estimates were pooled using Rubin’s rules. We used pooled likelihood ratio tests as implemented in the R package “mitml” (method D4) to assess the significance of the model terms [35]. Terms were tested according to the principle of marginality [36]. We calculated model-based marginal means using the R package “ggeffects” to illustrate the effect of NIHSS category, pre-stroke mobility limitation, comorbidities category, and FES-I score (quartiles) on the course of the LSA score. Continuous predictors were set at their respective means of the observed data during calculation of marginal means.

As an exploratory analysis, we elucidated typical trajectories of LSA by latent class growth analysis (LCGA) using the “lcmm” R package [37, 38]. Because of the small sample size, we opted to model time as linear continuous effect and did not allow heterogeneity within groups to keep the model parsimonious. We used the following criteria to select the optimal number of classes: BIC (smallest value), at least 5% of subjects in each class, mean posterior probability > 0.7 for all classes [39].

We performed univariate tests (ANOVA for continuous, Chi^2^ tests for categorical variables) for differences between the identified classes of trajectories. The *p*-values for the Chi^2^ tests were calculated by Monte Carlo simulations with 1e6 replicates. In addition to the independent variables used in the previous analyses, we tested for differences between the classes regarding LSA starting (*T*_0_; 3 months post stroke) value. We used the Benjamini–Hochberg approach to control the false-discovery rate and present the adjusted *p* values in addition to the unadjusted *p* values [40]. No imputation was performed for these exploratory analyses.

The level of significance was set at *p* ≤ 0.05; all tests were two-tailed. We used *R* version 4.2.1 for all statistical calculations.

## Results

### Participants

**Fig. 1:**
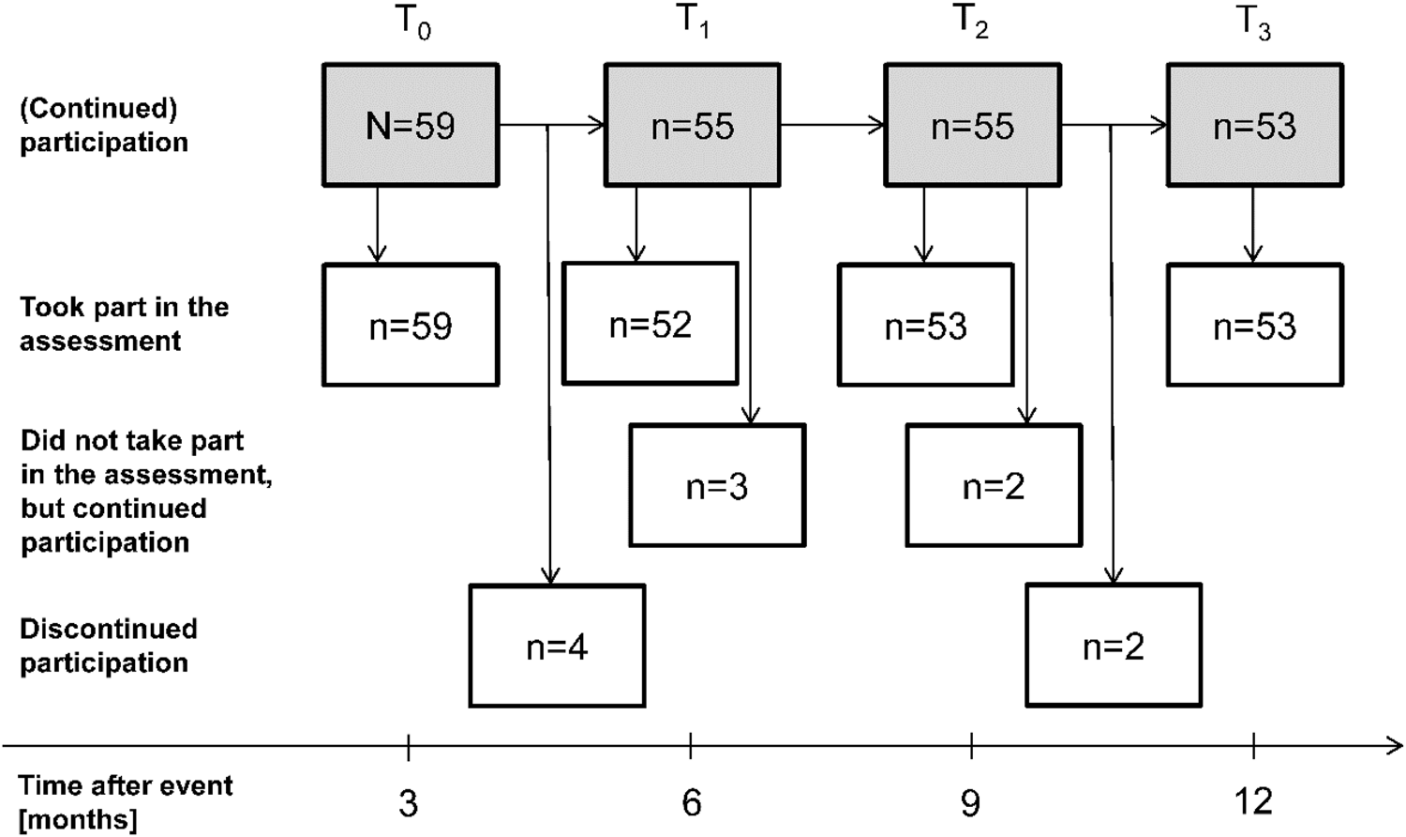
Flow of participants through the study

Participant characteristics (*N* = 59) are shown in Table 1**;** the flow of participants through the study is depicted in Fig. 1. On average, data collection took place (SD; range; time point) 92 (9; 74–110; *T*_0_) days; 177 (8; 167–205; *T*_1_) days; 268 (7; 259–295; *T*_2_) days; and 360 (8; 351–384; *T*_3_) days post stroke. Four participants dropped out between *T*_0_ and *T*_1_ for the following reasons: lack of interest (*n* = 2), health-related reasons (*n* = 1), and fear of COVID-19 infection (*n* = 1). Two participants dropped out between *T*_2_ and *T*_3_ for health-related reasons. In addition to the participants who had already dropped out at the respective point in time, three other participants did not take part in the *T*_1_ assessment for health-related reasons. Two participants did not take part in the *T*_2_ assessments due to vacation (*n* = 1) and for health-related reasons (*n* = 1).

**Table 1:** Participants' characteristics at *T*_0_

Characteristic	*N*	Category	n (%)	Mean	SD	Median	IQR
Sex	59	Female	20 (33.9)				
Age [years]	59			71.6	10.0	74	65, 78
NIHSS score	58	0	15 (25.9)				
		1	11 (19.0)				
		2	15 (25.9)				
		3	8 (13.8)				
		≥ 4	9 (15.5)				
Modified Rankin score	57	0	3 (5.3)				
		1	27 (47.4)				
		2	22 (38.6)				
		3	5 (8.8)				
Comorbidities^a^ [number]	59	≥ 2	44 (74.6)				
Pre-stroke mobility limitation	59	Yes (at least some difficulty walking 2 km or climbing stairs)	8 (13.6)				
Type of neighborhood	59	Rural	19 (32.2)				
		Suburban	20 (33.9)				
		Urban	20 (33.9)				
Availability of a car for personal use	59	Yes	32 (54.2)				
FES-I score	59			20.9	5.7	19	17, 22.5
Timed up-and-go [s]	59			10.1	4.2	9.0	7.4, 12.0

*SD* standard deviation, *IQR* interquartile range, *NIHSS* National Institutes of Health Stroke Scale, *FES-I* Falls Efficacy Scale-International Version

^a^As specified in the Self-Administered Comorbidity Questionnaire (SCQ)

### Description of life-space mobility

The descriptive analyses of life-space mobility in the first year after stroke (Table 2) showed a large variation of values around the mean and a wide IQR at each point.

**Table 2:** Descriptive analyses of life-space mobility 3, 6, 9 and 12 months after ischemic stroke

Measure	*T*_0_ (3 months)	*T*_2_ (6 months)	*T*_3_ (9 months)	*T*_4_ (12 months)
	*N* = 59	*n* = 52	*n* = 53	*n* = 53
LSA composite score				
Mean (SD)	69.3 (27.3)	72.3 (27.4)	79.3 (26.4)	77.8 (33.1)
Median [IQR]	72 [55.75, 92]	72 [53, 92]	86 [60, 100]	88 [49.5, 100]

*LSA* Life-Space Assessment, *SD* standard deviation, *IQR* interquartile range

### Factors associated with the course of life-space mobility

**Fig. 2:**
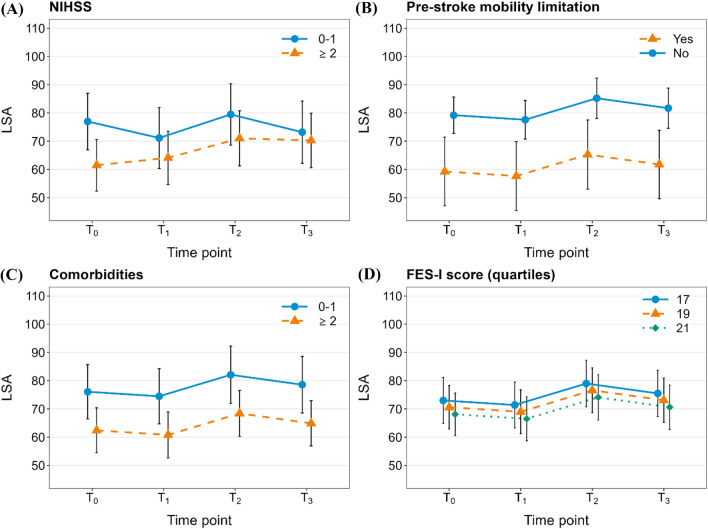
Marginal means (*N* = 59) illustrating the relationship between National Institutes of Health Stroke Scale (NIHSS) score category (0–1 vs ≥ 2) (**A**), pre-stroke mobility limitation (yes = at least some difficulty walking 2 km or climbing stairs vs no = no difficulty with either activity) (**B**); comorbidities category (< 2 vs ≥ 2 comorbidities) (**C**), as well as falls efficacy (quartiles of FES-I score) (**D**) and the course of the Life-Space Assessment (LSA) composite score 3 (*T*_0_), 6 (*T*_1_), 9 (*T*_2_) and 12 (*T*_3_) months after stroke

**Fig. 3:**
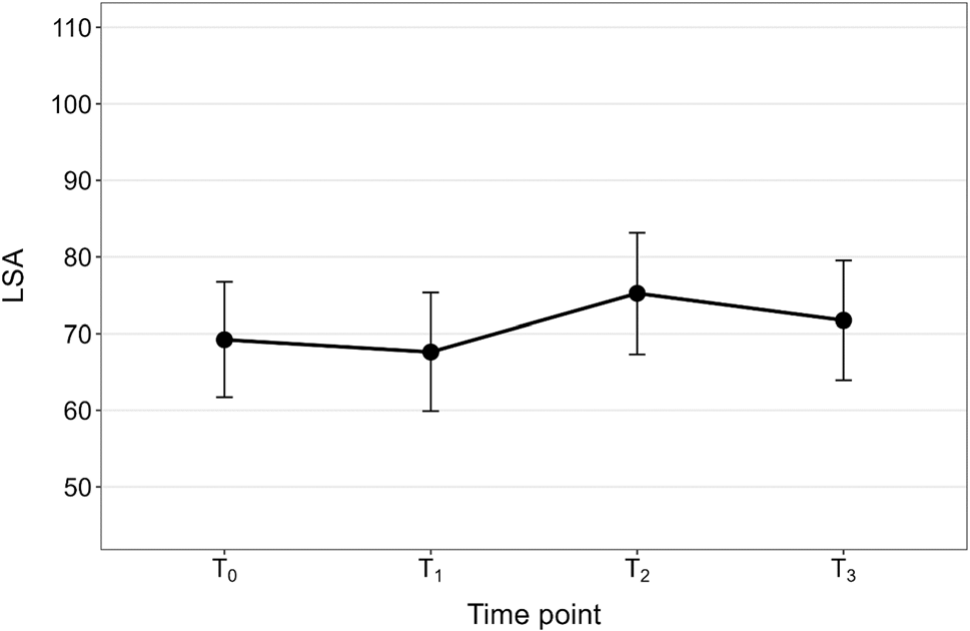
Marginal means illustrating the relationship between time point (*T*_0_ = 3, *T*_1_ = 6, *T*_2_ = 9, and *T*_3_ = 12 months after stroke) and Life-Space Assessment (LSA) composite score for the total sample (*N* = 59)

Based on LMMs with LSA score as outcome (Table 3), there was evidence that the following factors were associated with the course of LSA: pre-stroke mobility limitation, NIHSS category, FES-I score, and comorbidities category. The relationship between these factors and LSA is illustrated in Fig. 2A to D. The model revealed no evidence for an effect of time point on LSA (relationship depicted in Fig. 3).

**Table 3:** Results of linear mixed effects models with Life-Space Assessment (LSA) composite score as outcome (*N* = 59; imputed dataset)

Independent variables	*F*-value	Degrees of freedom	Beta coefficient (95% CI)^a^	*p* value^b^
Time point	2.12	3, 4456.3		0.095
* T*_1_ vs *T*_0_			2.5 (− 11.7, 16.7)	
* T*_2_ vs *T*_0_			11.2 (− 3.2, 25.5)	
* T*_3_ vs *T*_0_			10.3 (− 4.4, 19.7)	
Sex (male vs female)	0.61	1, 30985.6	7.7 (− 4.4, 19.7)	0.434
Age category (≤ vs > median)^c^	0.01	1, 2948.8	0.3 (-12.2, 12.8)	0.922
NIHSS category (0–1 vs ≥ 2)	3.99	1, 49590.6	− 15.5 (− 27.5, − 3.5)	**0.046**
Modified Rankin category (0–1 vs ≥ 2)	0.09	1, 2869.1	3.1 (− 10.2, 16.4)	0.762
Comorbidities category (< vs ≥ 2)^d^	8.79	1, 122958.1	− 13.7 (− 23.1, − 4.3)	**0.003**
Pre-stroke mobility limitation (yes vs no)^e^	10.08	1, 34009.2	19.9 (7.5, 32.4)	**0.001**
Type of neighborhood	2.25	2, 12528.5		0.105
Suburban vs rural			− 4.7 (− 13.9, 4.6)	
Urban vs rural			− 9.5 (− 19.2, 0.2)	
Availability of a car for personal use (no vs yes)	1.60	1, 622.2	5.2 (− 2.7, 13.1)	0.206
FES-I score	7.95	1, 813.8	− 1.2 (− 2.0, − 0.4)	**0.005**
Log-transformed TUG time [s]	2.80	1, 211.4	− 13.4 (− 26.7, − 0.1)	0.096
Interaction time point * sex	1.50	3, 5137.8		0.212
Interaction time point * age category	0.15	3, 4906.1		0.930
Interaction time point * NIHSS category	0.77	3, 3436.4		0.512
Interaction time point * modified Rankin category	0.51	3, 3096.8		0.676

*CI* confidence interval, *NIHSS* National Institutes of Health Stroke Scale, *FES-I* Falls Efficacy Scale-International Version, *TUG* timed up-and-go

^a^95% confidence intervals of beta-coefficients are based on* t*-distribution and pooled standard error using Rubin’s rules

^b^*p*-values are derived from likelihood-ratio tests; *p*-values ≤ 0.05 are bolded

^c^Median age was 74 years

^d^As specified in the Self-Administered Comorbidity Questionnaire (SCQ)

^e^At least some difficulty walking 2 km or climbing stairs vs no difficulty

### Typical trajectories

**Fig. 4:**
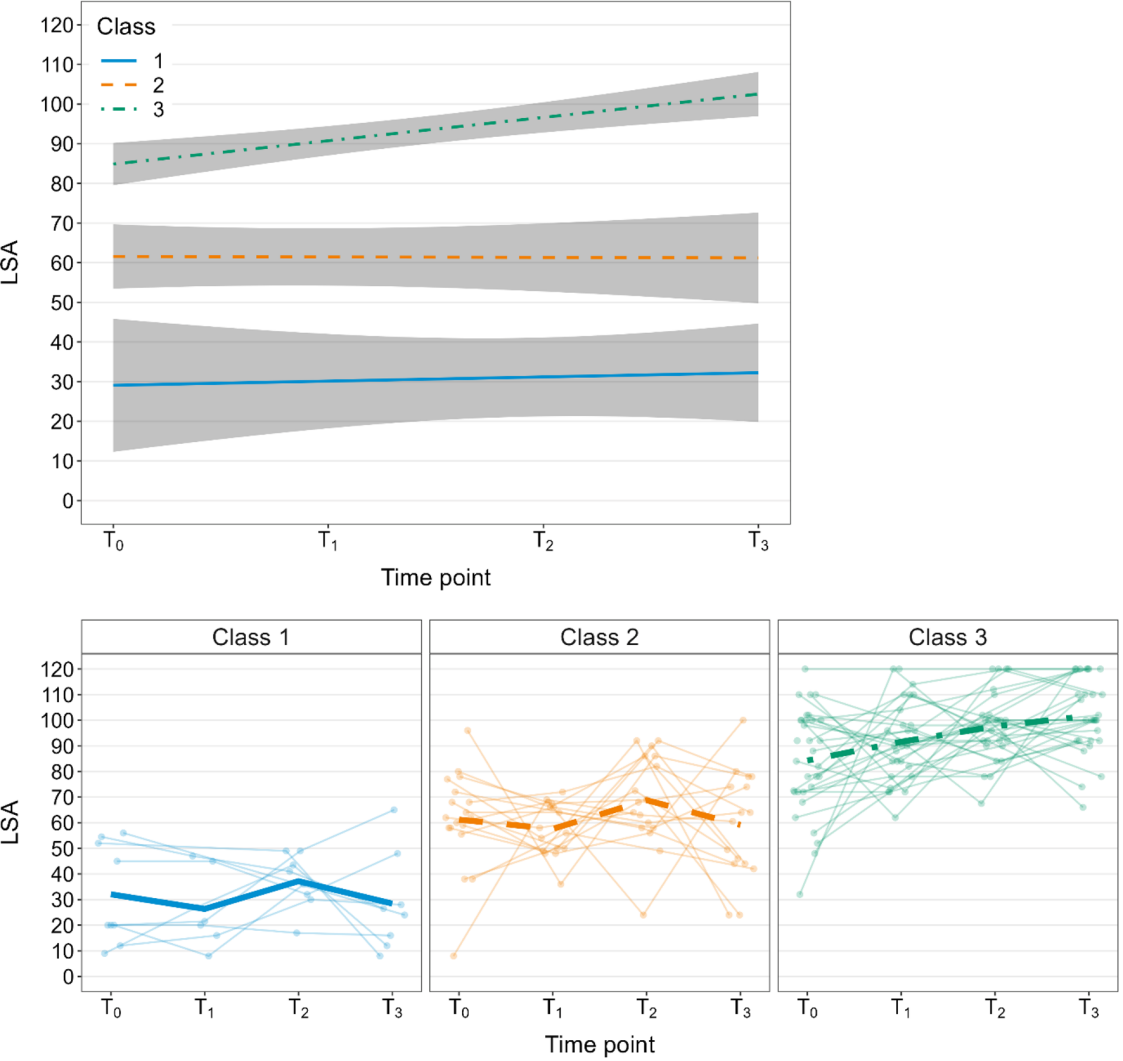
Illustration of the 3 typical trajectories of Life-Space Assessment (LSA) composite score revealed by latent class growth analysis (LCGA) (*N* = 59). The graph above shows the mean (modelled linearly over time) and the 95% confidence interval for every class; the graph below shows the data of each participant (thin lines) in the respective class and the empirical mean (thick lines) at 3 (*T*_0_), 6 (*T*_1_), 9 (*T*_2_) and 12 (*T*_3_) months post-stroke

The exploratory analysis of typical trajectories of LSA revealed three classes (Fig. 4) with *n* = 9 (class 1; “low stable”), *n* = 18 (class 2; “average stable”), and *n* = 32 (class 3; “high increasing”) participants, respectively. While for classes 1 (*p* = 0.767) and 2 (*p* = 0.956) we found no evidence for a change of LSA over time, there was evidence of an increase in LSA over time in class 3 (*p* < 0.001).

The univariate tests for differences between the classes (ANOVA or Chi^2^ tests respectively) revealed evidence (ie, unadjusted as well as adjusted *p* ≤ 0.05) that classes differed with regard to LSA starting value, pre-stroke mobility limitation, FES-I score, and log-transformed TUG time (Table 4). For the comorbidities category, only the unadjusted *p* value was ≤ 0.05 (adjusted *p* = 0.093). The mean LSA starting value (SD; median) was 32.1 (19.4; 20) in class 1; 61.2 (19.2; 62) in class 2; and 84.3 (20.5; 86) in class 3. In class 1, 4 out of 9; in class 2, 4 out of 14; and in class 3, 0 out of 32 had a pre-stroke mobility limitation. The mean (SD; median) FES-I score was 28.1 (9.0; 25) in class 1; 21.1 (3.9; 20) in class 2; and 18.8 (3.5; 17.5) in class 3. The mean (SD; median) log-transformed TUG time was 2.6 (0.4; 2.5) s in class 1; 2.3 (0.3; 2.4) s in class 2; and 2.1 (0.2; 2.0) s in class 3.

**Table 4:** Results of univariate tests (ANOVA for continuous and Chi^2^ tests for categorical variables) for differences between the three classes of trajectories

Variable	*N*	*n* class I/II/III	Test type	Test statistic	Degrees of freedom	Mean (95% CI) Class I	Mean (95% CI) Class II	Mean (95% CI) Class III	Un-adjusted *p* value^b^	Adjusted^a^ *p* value^b^
Sex	59	9/18/32	Chi^2^	3.21	NA				0.197	0.217
Age category (≤ vs > median)^c^	59	9/18/32	Chi^2^	4.10	NA				0.126	0.174
NIHSS category (0–1 vs ≥ 2)	59	9/18/32	Chi^2^	1.07	NA				0.625	0.625
Modified Rankin category (0–1 vs ≥ 2)	57	9/16/32	Chi^2^	4.63	NA				0.108	0.169
Comorbidities category (< vs ≥ 2)	59	9/18/32	Chi^2^	6.26	NA				**0.046**	0.093
Pre-stroke mobility limitation (yes vs no)^d^	59	9/18/32	Chi^2^	13.50	NA				**0.002**	**0.006**
Type of neighborhood (rural vs suburban vs urban)	59	9/18/32	Chi^2^	6.89	NA				0.148	0.180
Availability of a car for personal use (yes vs no)	59	9/18/32	Chi^2^	6.01	NA				0.051	0.093
FES-I score	59	9/18/32	ANOVA	13.23	2, 56	28.1 (24.9, 31.3)	21.1 (18.8, 23.3)	18.8 (17.2, 20.5)	** < 0.001**	** < 0.001**
Log-transformed TUG time [s]	58	9/17/32	ANOVA	11.36	2, 55	2.6 (2.4, 2.8)	2.3 (2.2, 2.5)	2.1 (2.0, 2.2)	** < 0.001**	** < 0.001**
LSA starting (T_0_) score	59	9/18/32	ANOVA	26.19	2, 56	32.1 (18.7, 45.4)	61.2 (51.8, 70.6)	84.3 (77.2, 91.3)	** < 0.001**	** < 0.001**

*NIHSS* National Institutes of Health Stroke Scale, *FES-I* Falls Efficacy Scale-International Version, *TUG* timed up-and-go, *ANOVA* analysis of variance, *NA* not applicable

^a^Benjamini–Hochberg approach to control the false-discovery rate

^b^*p*-values ≤ 0.05 are bolded

^c^Median age was 74 years

^d^At least some difficulty walking 2 km or climbing stairs vs no difficulty

## Discussion

In this prospective observational study of 59 patients, we found evidence that stroke severity, the presence of 2 or more comorbidities, pre-stroke mobility limitation, and falls efficacy affected the course of life-space mobility within the first year after stroke. Analyses of typical trajectories revealed three classes which can be described as “low stable”, “average stable”, and “high increasing”. Classes differed with regard to their starting LSA, pre-stroke mobility limitation, falls efficacy, and lower extremity physical function with a higher mean starting LSA, lower prevalence of pre-stroke mobility limitation, higher mean falls efficacy, and better mean lower extremity physical function in the “high increasing” class.

Median LSA values of our sample ranged between 72 (IQR 55.75–92) at 3 months and 88 (IQR 49.5–100) at 12 months after stroke, respectively; these values were markedly higher than those reported by Tsunoda et al. in patients at a median time after stroke of 75 (IQR 19–120) months (median LSA 48.0; IQR 36.0–67.5) [17]. Despite some similarities between the two samples (our sample vs Tsunoda et al.), including a comparable age and sex distribution and a high median level of functional independence of both samples, comparability is limited by discrepancies in inclusion criteria (our study: ability to walk for 20 m vs Tsunoda et al.: ability to walk for 5 m) and in starting point and length of the time periods studied (our study: first year after event vs Tsunoda et al.: a period of 2 years starting at a median of 75 months after stroke). The much earlier starting point and the relatively high burden (four assessments) within the first year after stroke might have led to a selection of healthier and fitter participants in our study; indicated by a markedly better lower extremity function in our sample (Tsunoda et al.: median comfortable walking speed of 0.66 m/s vs. our sample: median TUG time of 9.0 s). Median LSA values of our sample are comparable to values found in population-based studies in community-dwelling older adults with median scores typically between 55 and 75 [10–12, 41]. While in our sample, there was no evidence of a change in LSA over time in the multivariate analyses, Tsunoda et al. reported a significant decline within the 2-years follow-up period [17]. We additionally conducted an exploratory LCGA, indicating that there was 1 group of participants (class 3; “high increasing”; about half of the sample) with a high LSA starting value who seemed to be able to increase their LSA even further in the first year. The other groups with lower LSA starting values remained stable.

The only existing study [17]—to the best of our knowledge—on factors associated with longitudinal changes in LSA in patients after stroke identified comfortable gait speed and age as independent factors in multiple LMMs. The authors found no evidence for effects of sex, time after event, type of stroke (ischemic vs hemorrhagic), presence of diabetes, functional independence, or cognition. Positive relationships between lower extremity physical function and life-space mobility are well-documented in the general population [42, 43] and have also been demonstrated by a number of cross-sectional studies in post-stroke patients. As an example, a cross-sectional study by Tashiro et al. [18] in 46 community-dwelling individuals with a median time of 49.5 months (IQR 32–90.5) post-event showed a significant association between maximum walking speed (m/s) and LSA score in a multiple regression analysis (coefficient *β* = 12.85; 95% CI 2.46–23.23; *p* = 0.017). In another cross-sectional study in 112 people after stroke (average time post-event 73.6; SD 57.4 months), lower extremity physical function, assessed by the Five Times Sit-to-Stand Test (lower values indicate better function), correlated negatively with LSA in unadjusted analyses (Spearman correlation coefficient *r* = − 0.42; *p* < 0.001) [19]. A longitudinal study aiming to predict LSA scores 2 months after discharge from inpatient rehabilitation after stroke based on parameters assessed at discharge identified a TUG time of < 15 s as being predictive of higher LSA scores (*p* < 0.0001) [44]. In our study, the multivariable LMMs did not reveal evidence for an effect of lower extremity physical function (measured by TUG) on the course of LSA. However, those belonging to the class (identified by LCGA) with high starting LSA value and increase of LSA over time (class 3; “high increasing”) had better TUG values than their counterparts in the other classes (classes 1 and 2; “low stable” and “average stable”). Previous cross-sectional analyses of our sample at 3 months post-stroke showed that log-transformed TUG time was negatively associated with objective life-space measures assessed by Global Navigation Satellite System (GNSS) over a 1-week period, such as the maximum distance from home and the convex hull area (the smallest convex polygon on a map enclosing all GNSS fixes) [45].

Stroke severity (measured by NIHSS) has repeatedly been shown to be one of the main predictors of functional limitations and disability after stroke [5, 46]. Physical, cognitive as well as perceptual deficits associated with stroke may compromise the patients’ ability and confidence to navigate through their community environment and thereby limit their social participation [47]. Our data suggest that even within a sample of patients with predominantly mild stroke severity (95% with a NIHSS score of ≤ 5), the severity (NIHSS score of 0–1 vs ≥ 2) affected the course of life-space mobility in the first year after stroke—with better LSA scores in those belonging to the NIHSS 0–1 category. The observed gain in LSA in those with higher NIHSS score (≥ 2) (Fig. 2A) may—at least partly—reflect the recovery of their neurological deficits.

Our findings also suggest that the presence of comorbidities was associated with the course of LSA after stroke—with higher LSA scores in those with fewer (0–1 vs ≥ 2) comorbidities. To the best of our knowledge, there are no previous reports on the association between comorbidities and life-space mobility in patients after stroke; however, comorbidities have repeatedly been shown to be prognostic of functional recovery, participation in life situations and survival post-stroke [4, 47–50]. The presence of comorbidities in patients after stroke may affect life-space mobility through various biopsychosocial pathways; besides potentially causing additional physical, cognitive or perceptual deficits, they may also contribute to an increased psychological distress [51] as well as physical and mental fatigue [52].

In our study, falls efficacy was positively associated with life-space mobility after stroke. This is in line with previous findings that the FES-I score at discharge from primary rehabilitation predicts the LSA score 2 months after discharge [44]. Our findings are also in line with the abovementioned cross-sectional study by Tashiro et al. [18] in individuals post-stroke, which showed a significant association between FES-I score and LSA in a multiple regression analysis (coefficient *β* = − 0.303; 95% CI − 0.590 to − 0.015; *p* = 0.039). In contrast to this study with a median FES-I score of the participants of 43.5 (IQR 34–59), the median FES-I score of our sample was much lower (median 19; IQR 17–22.5), indicating a lower median concern of falling. This illustrates that even slight deteriorations in perceived self-efficacy to perform daily activities without falling may lead to restrictions of life space and social participation in patients after stroke.

Our data showed that self-reported mobility limitation in the week before the event was associated with the course of life-space mobility in the first year after the event. When clinicians treat patients after their first stroke, it can be difficult to differentiate between potentially pre-existing limitations and limitations caused by the event itself; especially considering that within the general population aged 75–84, 23% are unable to walk half a mile and 15% are unable to climb stairs [53]. In order to better predict the potential for recovery, it may be helpful to routinely apply a retrospective assessment of pre-existing mobility limitations. It should however be considered that such measures may be affected by recall bias and may therefore not be useful in patients with severe cognitive impairment (who were excluded from participation in our study).

### Limitations and strengths

The limited sample size meant that the selection of covariables for the statistical analyses was not exhaustive. It is therefore possible that other relevant determinants or confounders were overlooked, leading to residual confounding. The inclusion of a relatively high number of independent variables in relation to the rather small sample size increased the chance of missing an existing association; ie, the fact that our study did not find evidence for associations between some of the independent variables and the outcome should be interpreted with great care [54]. Furthermore, the small sample size limits the generalizability of the results. Data collection took place between January 2020 and February 2022, ie, within a time period that was affected by social distancing recommendations due to the COVID-19 pandemic. Hence, the overall level of life-space mobility of our participants, particularly of those with higher age, may have been reduced within this time period [55]. Strengths of the study include the longitudinal design with repeated measurements at clearly defined time points after stroke.

## Conclusion

Routinely assessing pre-stroke mobility limitation, LSA starting value and falls efficacy—in addition to traditional routine parameters such as the NIHSS and comorbidities—may help clinicians to identify patients at risk of a lack of progress in regaining life-space mobility. Falls efficacy can potentially be modified and improved through targeted rehabilitative measures.

## Data Availability

The data that support the findings of this study are available from the corresponding author, TH, upon reasonable request.
